# Twelve mutations, three trials, and five different labels: PARP inhibitors regulatory inconsistencies in prostate cancers

**DOI:** 10.1016/j.eclinm.2025.103725

**Published:** 2025-12-30

**Authors:** Sahar Barjesteh van Waalwijk van Doorn-Khosrovani, Hans M. Westgeest, Timothée Olivier

**Affiliations:** a Department of Medical Oncology, Leiden University Medical Centre, Albinusdreef 2, 2333 ZG, Leiden, the Netherlands; b CZ Health Insurance, Ringbaan West 236, 5038 KE, Tilburg, the Netherlands; c Department of Internal Medicine, Amphia Hospital, Molengracht 21, 4818 CK, Breda, the Netherlands; d Department of Oncology, Geneva University Hospital, 4 Gabrielle-Perret-Gentil Street, 1205, Geneva, Switzerland

**Keywords:** PARP-Inhibitors, mCRPC, Homologous recombination repair, Homologous recombination repair deficiency, Regulatory inconsistencies, Prostate cancer

## Abstract

Three different PARP (Poly (ADP-ribose) Polymerase)-inhibitors have been approved in combination with androgen receptor pathway inhibitors (ARPIs) for the treatment of metastatic castration-resistant prostate cancer (mCRPC). Regulatory authorities, however, have divergent opinions. Although the US Food and Drug Administration (FDA) has limited approval of two PARP-inhibitors to patients with *BRCA* mutations or other alterations in homologous recombination repair (HRR) genes, the European Medicines Agency (EMA) has approved the indication for the overall mCRPC population, irrespective of HRR status. Of all trials, only MAGNITUDE, evaluating niraparib and abiraterone, led to aligned conclusions from both the EMA and FDA, as its design effectively identified the subgroup most likely to benefit. The discrepancies observed in the assessment of the other two trials stem from limitations in their designs. A key issue with PROpel is the lack of patient stratification based on known biomarkers, and the subgroup analysis is underpowered. In TALAPRO-2, although an enriched cohort is included, combining these data with the all-comers cohort results in a potentially misleading conclusion. Currently, there is a need for harmonisation in biomarker-driven trial designs and the definition of homologous recombination repair deficiency (HRD). Access to biomarker and clinical data from all PARP-inhibitor trials would allow researchers to clarify the impact of different HRR mutations on outcomes.

## Introduction

The development and regulatory assessment of three PARP (Poly (ADP-ribose) Polymerase) inhibitors in combination with Androgen Receptor Pathway Inhibitors (ARPI) for the first-line treatment of metastatic castration-resistant prostate cancer (mCRPC) illustrates important differences between American and European regulators, and varying methods of analysing therapeutic benefit by lumping or splitting biomarker categories.^1^

Prostate cancer may involve mutations in homologous recombination repair (HRR) genes (HRRm), which can lead to homologous recombination repair deficiency (HRD). Those are usually grouped in two buckets, *BRCA1* and *BRCA2* mutations (*BRCA*m) makes one group, and non-*BRCA* mutations (non-*BRCA* HHRm) constitute the other group, with mutations individually less frequent: *ATM*, *ATR*, *CHECK1*, *CHEK2*, *FANC, MRE11A, PALB2*, *RAD51C* and others.^2^ Finally, most prostate cancer patients do not have any of these mutations and are considered homologous recombination repair proficient.

**Fig. 1:**
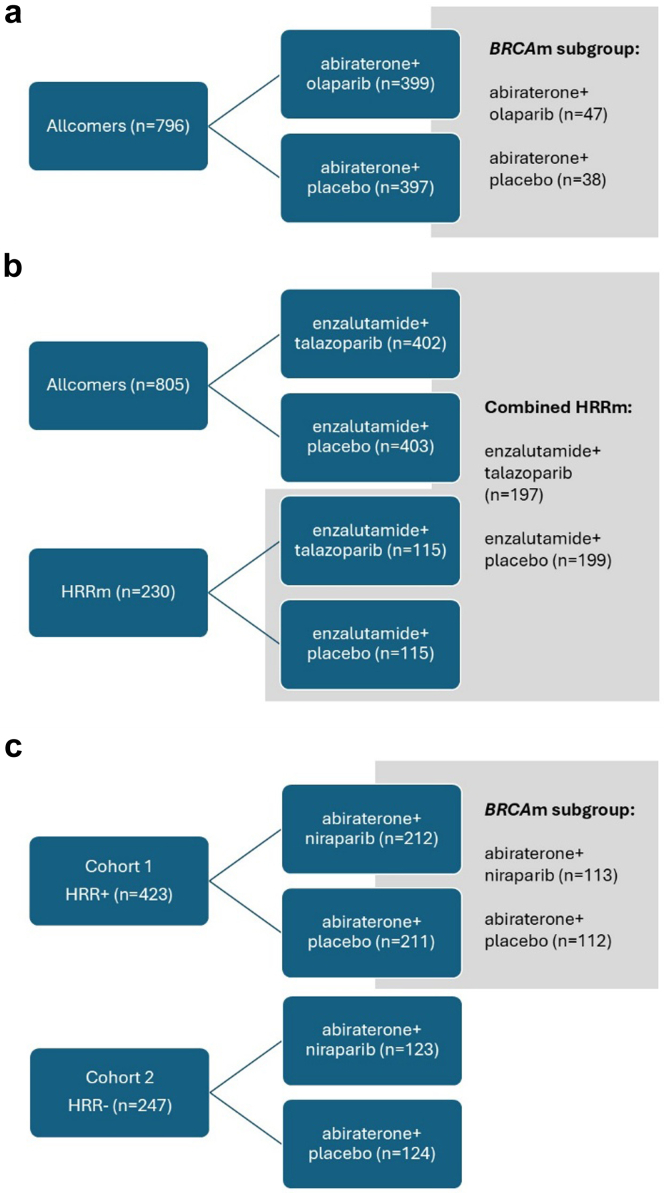
The design of trials studying the combination of a PARP (Poly (ADP-ribose) Polymerase) inhibitor and an androgen receptor pathway inhibitors (ARPI) in metastatic castration-resistant prostate cancer (mCRPC): a. PROpel b. TALAPRO-2 c. MAGNITUDE; and the five diverse cohorts that have been authorised by the Food and Drug Administration (FDA) and the European Medicines Agency (EMA). Proportions approximate the percentage of patients in each group (d). HRRm: mutation in Homologous Recombination Repair genes; FDA: Food and Drug Administration; EMA: European Medicines Agency.

Three different clinical trials have been conducted in this space,^3^, ^4^, ^5^, ^6^ leading to 5 different approved indications between the US Food and Drug Administration (FDA) and the European Medicines Agency (EMA; Fig. 1, Table 1). For niraparib, the US and European label are similar and most restrictive– for patients with *BRCA*m only– niraparib plus abiraterone and prednisone is authorised front line.^7^^,^^8^ For both talazoparib^9^ and olaparib,^10^ the EMA has granted broader approvals than the FDA. FDA restricts these drugs to HRRm^11^ and *BRCA*m^12^ respectively, while the EMA expands the indications to the overall population, regardless of HRR status.

**Table 1:** Regulatory approvals and clinical efficacy across PROpel, TALAPRO-2, and MAGNITUDE trials.

	PROpel	TALAPRO-2	MAGNITUDE
FDA approval	Population: *BRCA*m; Date of approval: May 2023	Population: HRRm; Date of approval: June 2023	Population: *BRCA*m or suspected *BRCA*m; Date of approval: August 2023
Clinical efficacy data for the population authorised by FDA^a^	Median rPFS NR vs 8 m (HR 0.24; 95% CI 0.12–0.45) and OS NR vs 23 m (HR 0.30; 95% CI 0.15–0.59)	Median rPFS NR vs 13.8 (HR 0.45; 95% CI 0.33–0.61) and OS NR vs 33.7 (HR 0.69; 95% CI 0.46–1.03)	Median rPFS 16.6 vs 10.9 m (HR = 0.53, 95% CI 0.36–0.79) and OS 30.4 vs 28.6 months (HR 0.79; 95% CI 0.55–1.12)
EMA approval	Population: overall, regardless of HRR/*BRCA* status; Date of approval: November 2022	Population: overall. regardless of HRR/*BRCA* status, if chemotherapy is not indicated; Date of approval: November 2023	Population: *BRCA*m; Date of approval: February 2023
Clinical efficacy data for the population authorised by EMA^a^	Median rPFS 24.8 vs 16.6 (HR 0.66; 95% CI 0.54, −0.81) and OS 27.6 vs 26.3 m (HR 0.83; 95% CI 0.66–1.03)	Median rPFS (Cohort 1) NR vs 21.9 (HR 0.63; 95% CI 0.51–0.78) and OS NR vs 38.2 (HR 0.84; 95% CI 0.67–1.04)	Median rPFS 16.56 vs 10.87 m; HR = 0.53, 95% CI 0.36–0.79) and OS 29.27 vs 28.55 months (HR 0.88; 95% CI 0.58–1.34)

FDA: US Food and Drug Administration; EMA: European Medicines Agency; HRR: Homologous Recombination Repair; HRRm: mutations in HRR genes; HR: Hazard Ratio; CI: Confidence Interval; rPFS: radiographic Progression-Free Survival; OS: Overall Survival; NR: Not Reached.

a Data available to the agency at the time of evaluation.

Some of these differences are based upon different statistical philosophies. Some statisticians argue that one should have statistically persuasive data to treat a subgroup differently before precluding that subgroup from receiving a therapy– and this view appears to dominate in Europe. In contrast, some clinicians may argue that ascertaining the benefit of a therapy must take into account a biologic understanding and pretest probability of benefit, and it is unlikely that targeted therapies work when the target is absent, and this philosophy appears to be prevalent within the FDA.

Ultimately, for patients and doctors, there remains massive uncertainty. Who benefits from these therapies? Does it vary by mutation? Are there unexplored differences– for example not all non-*BRCA* HRDm behave similarly– i.e. *ATM* is different than *RAD51*? And how will clinical trials sort this out? In the meantime, there is considerable risk that toxic, costly medications are broadly administered to prostate cancer patients who have *a priori* no possibility of benefit. In this viewpoint, we describe the clinical trial landscape of these therapies. We explain the available and disputing subgroup analyses and review the regulatory decisions. Finally, we conclude with a suggested post-marketing framework for achieving what patients and doctors want most: figuring out the right drug to give the right patient at the right time.

## PROpel, all-comer design with no stratification or prespecified alpha-controlled analysis

The PROpel trial^3^^,^^4^ randomly assigned 796 patients with mCRPC (1:1) to olaparib or placebo with abiraterone (plus prednisone or prednisolone) between October 2018 and January 2020 (Fig. 1a). The trial adopted an all-comer design with no stratification or prespecified alpha-controlled analysis for *BRCA*m/HRRm, despite the EMA's recommendation to do so.^10^

The trial met its primary endpoint, with a median radiographic progression-free survival (rPFS) of 24.8 months in the olaparib arm vs 16.6 months in the control arm [HR 0.66, 95% CI 0.54–0.81; p < 0.001]. However, there was no statistically significant difference in overall survival (OS) between the two groups. Both regulatory agencies, suspected that the potential benefit of adding olaparib to abiraterone might be driven by the subgroup of patients with *BRCA*m (n = 85 [11%]; rPFS HR 0.24, 95% CI 0.12–0.45; overall survival [OS] HR 0.30, 95% CI 0.15–0.60). No significant effect was observed in the non-*BRCA*m subgroup (n = 427; rPFS HR 0.85, 95% CI 0.66–1.11; OS HR 1.06, 95% CI 0.81–1.39) and there were concerns about unnecessary exposure and side effects.^10^^,^^13^^,^^14^

It is noteworthy that for 36% (284/796) of the patients, the *BRCA*m status was undetermined, and therefore any effect could still be attributed to unidentified mutations. Another similar but smaller exploratory trial, Study 8,^15^ also showed a lack of benefit and potential OS detriment in the non-*BRCA*m subgroup (OS HR 2.77, 95% CI 1.06–8.06).^13^

In general, if a positive trial demonstrates benefit in a biomarker-positive subgroup, FDA guidance allows for approval exclusively for that population.^16^ Based on this guidance, the FDA restricted the indication to deleterious or suspected deleterious *BRCA*m.^13^ In contrast to the FDA, the EMA authorised a broad label for the overall population, regardless of *BRCA*m or HRRm.^10^ Their reasoning was that the company had not followed the recommendation to stratify and power the subgroups based on HRR status, and therefore the results in the subgroups should be interpreted with caution, as they are exploratory.

## TALAPRO-2, all-comers design with stratification and an enriched cohort

TALAPRO-2^5^ was all-comers, randomised (1:1), double-blind, placebo-controlled trial in mCRPC, comparing talazoparib plus enzalutamide with placebo plus enzalutamide in patients with and without HRRm. The trial accrued 805 patients between January 7, 2019, and September 17, 2020 (Fig. 1b). The difference with PROpel is that the HRRm status was prospectively determined to allow stratification of patients. This cohort was followed by a second cohort, which enrolled only patients with HRRm mCRPC (n = 230), and outcomes were analysed in the combined HRRm population across both cohorts.

In TALAPRO-2, both independent primary endpoints of rPFS in the all-comers cohort (HR 0.63, 95% CI 0.51–0.78; p < 0.0001) and rPFS in the combined HRRm population (HR 0.45, 95% CI 0.33–0.61; p < 0.0001) were met. The rPFS HR for non-*BRCA* HRRm was 0.72 (95% CI 0.49–1.07) and the OS HR was 0.71 (95% CI 0.43–1.18). In the non-HRRm/unknown subgroup the rPFS HR was 0.70 (95% CI 0.54–0.89).^11^

As in PROpel, the FDA decided that the magnitude of the rPFS benefit, the side effects, and immature OS data were insufficient to support authorisation in non-HRRm. A broad authorisation would result in a large group of patients being exposed to the added toxicity of combination therapy for an extended period, regardless of talazoparib's benefit.^11^

Once again, in contrast to the restricted FDA label, the EMA authorised the indication for the overall population. Their reasoning was that the established rPFS gain and the absence of detrimental effects on OS in any of the subgroups (although the data are not yet mature) support approval for the all-comers population, regardless of HRR status.^9^ Recently, the updated results from TALAPRO-2 with a median follow-up of 44·2 months^17^ and 52.5 months^18^ were published. The final OS results show a significant improvement with talazoparib plus enzalutamide (HR 0.80, 95% CI 0.66–0.96; p = 0.016). Overall survival was significantly better in HRRm subgroup (HR 0.55, 95% CI 0.36–0.83; p = 0.0035) but not significantly different in non-HRRm or unknown patients (HR 0.88, 95% CI 0.71–1.08; p = 0.22).^18^

## MAGNITUDE, a biomarker-stratified design with an enriched HRRm cohort

MAGNITUDE,^6^ enrolled between February 2019 and June 2021^8^ and prospectively assigned mCRPC patients to either Cohort 1 (HRRm, n = 423) or Cohort 2 (non-HRRm, n = 247) based on biomarker status, followed by randomisation (1:1) to receive either niraparib with abiraterone or placebo with abiraterone (plus prednisone; Fig. 1c). For Cohort 2, originally, 600 patients were planned; however, results from a pre-specified futility analysis suggested no clinical benefit, and the cohort was stopped early.

As the greatest effect was expected in *BRCA*m, a protocol amendment was implemented in 2020, approximately one year after the start of the trial, to modify the statistical analysis plan. The *BRCA*m subgroup was assigned as the primary efficacy population to be analysed first. If statistical significance was reached in this subgroup, the entire Cohort 1 population would then be tested. Median rPFS in the *BRCA*m subgroup was significantly longer in the niraparib group vs the control group (19.5 months vs 10.9 months; HR 0.55, 95% CI 0.39–0.78; p = 0.0007). In the overall Cohort 1, median rPFS was also significantly longer in the niraparib group compared with the control group (16.7 months vs 13.7 months; HR 0.76, 95% CI 0.60–0.97; p = 0.0280).^6^ Nevertheless, both the FDA^7^ and EMA^8^ restricted the label to the *BRCA*m population, because this subgroup clearly benefited from the combination therapy. This decision was further supported by the lack of a significant effect in non-*BRCA* HRRm patients (rPFS HR 0.99, 95% CI 0.67–1.44) and the potential detrimental effect on overall survival (OS HR 1.13, 95% CI 0.77–1.64).^9^

## Inconsistencies in approvals are due to trial design

Of the three trials, only MAGNITUDE led to the same conclusion from both the EMA and FDA, as its design effectively identified the benefiting subgroup (Fig. 1d). The discrepancies observed in the assessment of the other two trials stem from differences in their design. A key problem with PROpel is that it does not stratify patients based on known biomarkers, and the subgroup analysis is underpowered. In TALAPRO-2, although there is an enriched cohort, combining the data with the all-comers cohort (Cohort 1) leads to a misleading conclusion.

One significant pitfall is the sequential assessment of the biomarker-positive subgroup and the overall population. If the effect in the biomarker-positive subgroup is large enough, it can lead to overall trial positivity and potentially unjustified approval. In such cases, alpha (the significance level) must be adjusted to control for Type I error. Otherwise, there is a high risk of recommending an ineffective therapy for biomarker-negative population. Harmonising trial designs and endpoints, in collaboration with regulators, can reduce discrepancies in approvals and provide a clearer understanding of treatment efficacy.

## Uncertain benefits of combination therapies

Granting a broad label for a trial with design flaws disincentivises companies from conducting informative trials and raises clinical concerns, especially when dealing with combination therapy. In contrast to monotherapy, where ineffective drugs are discontinued, PARP inhibitor use will continue simply due to the effectiveness of ARPIs.^11^^,^^13^ This over-treatment comes with increased toxicity and higher healthcare costs. Furthermore, a combination therapy should preferably be compared to the sequential use of the individual drugs to assess its true benefit. In this case, it would have been desirable to offer patients in the *BRCA*m/HRRm subgroup of the control arm a PARP inhibitor upon first radiographic progression.

Finally, there is the issue of control arm.^19^ In these studies, patients in the control arm who had previously received an ARPI arguably received substandard care by being assigned another ARPI.^20^ The EMA also raised concerns about patients with visceral disease who had not received docetaxel but had been treated with an ARPI, and therefore restricted the indication to those for whom chemotherapy is not clinically indicated.^8^, ^9^, ^10^

## Post-market considerations: harmonisation in the definition of HRD

Currently, there is a need for harmonisation in the definition of HRD, as it is not consistent across trials.^11^^,^^21^ For example, the PROpel trial did not consider *ATR*, *NBN*, *FANCA* and *MRE11A* genes^4^ whereas TALAPRO-2 included *MLH1*, a mismatch repair gene, which is not part of the HRR pathway.^11^ In TALAPRO-2, the greatest benefit was seen in patients with *BRCA* and *CDK12* mutations. Remarkably, the rPFS improvement in the non-*BRCA* HRRm subgroup was not statistically significant, but the FDA approved it nonetheless, given the limited interpretability due to small sample size.^11^

Ideally, researchers should be given access to both biomarkers and clinical data from all PARP inhibitor trials. Aggregating data can increase statistical power and help us better understand how different HRRm impact treatment outcomes. In addition, it is important to explore the potential impact of other concomitant genomic aberrations^22^ and to use HRD assays alongside mutations in key HRRm genes to provide a functional readout of homologous recombination status. HRD scores, defined as the sum of loss-of-heterozygosity (LOH), telomeric allelic imbalance and large-scale state transitions, represent promising biomarkers whose clinical utility needs to be assessed in prospective studies.^23^ A retrospective genomic analysis of TALAPRO-1, a talazoparib monotherapy trial in heavily pretreated castration-resistant prostate cancer with mutations in DNA damage response HRR genes, shows that the objective response rate (ORR) is significantly higher in patients with high genomic loss-of-heterozygosity (gLOH) compared with low [53.3% (16/30) vs 12.0% (3/25); p = 0.0017]. The median OS was also higher in gLOH-high patients (23.7 months vs 18.7 months [HR = 0.92 (95% CI 0.52–1.64)]. However, before these findings can guide treatment recommendations, the independent contribution of gLOH must be confirmed in larger studies.^22^

## Conclusion

The dossier of PARP inhibitors in mCRPC highlights how divergent and inconsistent trial designs may lead to different conclusions. Conversely, a well-designed trial ensures that patients, clinicians, regulators, and payers are not left in uncertainty. An optimal trial in this setting would prospectively determine HRR status using a standardised definition, followed by stratified randomisation into biomarker-defined cohorts (e.g., *BRCA1/2*, non-BRCA HRRm, and non-HRRm). Each cohort would be independently powered with pre-specified alpha allocation to enable definitive conclusions, and to avoid the risk of one cohort driving the benefit of a wider cohort (“adjacent subgroups” design instead of “nested subgroups” design). Importantly, the control arm should reflect contemporary standard of care, allowing a fair comparison between combination therapy vs optimised sequential use. Even though no trial is perfect, these funding principles would limit the risk that a trial is ultimately non-informative stemming from its very inception. Regulatory policies should encourage more harmonised trial designs, not only through scientific advice but also by providing explicit guidance, including on the use of appropriate control arms. Consistent biomarker definitions, even if not achievable at the outset of development, should be calibrated and harmonised post-approval. Only in this way can we ensure the development of valuable treatments for well-defined patient populations.

## Contributors

SBvWvD contributed to the conception and design of the study and drafted the initial manuscript. All authors verified the data, contributed to its interpretation and analysis, critically reviewed and revised the manuscript, and approved the final version for publication.

## Declaration of interests

SBvWvD's institution received research funding from Horizon Europe Cancer Mission, EC grant agreement no. 101104269 (PRIME-ROSE), unrelated to this work. The following organisations have (partially) covered travel expenses for SBvWvD's presentations (unrelated to this work): CIRS, CDDF, Precision Medicine Forum, Partners for Patients NGO, OMICO, EMA, SYNC 2024, EP PERMED, ISPOR, EU-X-CT, Remedi4ALL, VISION EUROPE 2030, and EU Commission/EU HTA. Timothée Olivier and Hans M. Westgeest have no financial or non-financial conflicts of interest to report.
